# Analytic Calculation of Finite-Population Reproductive Numbers for Direct- and Vector-Transmitted Diseases with Homogeneous Mixing

**DOI:** 10.1007/s11538-014-9950-x

**Published:** 2014-04-23

**Authors:** Lindsay Keegan, Jonathan Dushoff

**Affiliations:** Department of Biology, McMaster University, Hamilton, ON Canada

**Keywords:** Basic reproductive number, Transmission factors, Malaria, Modeling, Vector-borne diseases

## Abstract

The basic reproductive number, $\mathcal {R}_{0}$, provides a foundation for evaluating how various factors affect the incidence of infectious diseases. Recently, it has been suggested that, particularly for vector-transmitted diseases, $\mathcal {R}_{0}$ should be modified to account for the effects of finite host population within a single disease transmission generation. Here, we use a transmission factor approach to calculate such “finite-population reproductive numbers,” under the assumption of homogeneous mixing, for both vector-borne and directly transmitted diseases. In the case of vector-borne diseases, we estimate finitepopulation reproductive numbers for both host-to-host and vector-to-vector generations, assuming that the vector population is effectively infinite. We find simple, interpretable formulas for all three of these quantities. In the direct case, we find that finite-population reproductive numbers diverge from $\mathcal {R}_{0}$ before $\mathcal {R}_{0}$ reaches half of the population size. In the vector-transmitted case, we find that the host-to-host number diverges at even lower values of $\mathcal {R}_{0}$, while the vector-to-vector number diverges very little over realistic parameter ranges.

## Introduction

The basic reproductive number, $\mathcal {R}_{0}$, measures the expected number of new infections that can be traced back to a single infectious individual in an otherwise totally susceptible population. The concept of $\mathcal {R}_{0}$ provides a foundation for evaluating when infectious diseases can spread in a population, what factors determine disease incidence, and when interventions can eliminate disease (Dietz 1993; Heffernan et al. 2005). Its foundations go back over a century (Ross 1910; Kermack and McKendrick 1927).

In a study of malaria reproductive numbers, Smith et al. (2007) pointed out that classical calculations of $\mathcal {R}_{0}$ implicitly assume infinite host population sizes, and are hard to interpret when $\mathcal {R}_{0}$ approaches or exceeds the population size. They introduced the idea of measuring the typical number of new infections per infectious individual for a disease invading *a finite host population*. These finite-population reproductive numbers account for the fact that some individuals get bitten by multiple mosquitoes, at random, and absorb some of the infections. However, like classic calculations of $\mathcal {R}_{0}$, Smith et al. (2007) are only interested in the initial spread of infection; their estimates of these reproductive numbers ignore longer term depletion of susceptibles. They used simulations to estimate the vector-to-vector and host-to-host reproductive numbers, which they called $\mathcal{{Z}}_0(H)$ and $\mathcal{{R}}_0(H)$, respectively, and ask how these reproductive numbers change when vector biting is heterogeneous—where some hosts are more attractive to mosquitoes than others. They showed that in the case of finite-sized populations, unlike the infinite-population case, the number of vectors infected per vector is not necessarily the same as the number of hosts infected per host, and suggested that measuring $\mathcal{{Z}}_0(H)$ and $\mathcal{{R}}_0(H)$ could be informative for understanding the effects of different control measures.

Other studies (Keeling and Grenfell 2000; Ross 2011) have done similar work on directly transmitted diseases using stochastic models, here we use a next-generation framework to explore the impact of finite-population size on both directly transmitted and vector-borne diseases.

Here, we take a step towards better understanding of these “finite-population reproductive numbers” by calculating them analytically for homogeneous mixing between hosts (or hosts and vectors). We consider both directly transmitted and vector-transmitted diseases. For directly transmitted diseases, we calculated the average number of hosts infected by a single infectious host, which we call $\mathcal {R}(N)$. In the latter case, we calculate separate finite-population reproductive numbers for transmission from host species (via the vector) back to the host species, and for the vector species (via the host) back to the vector species which we call $\mathcal {R}(H)$ and $\mathcal {Z}(H)$ respectively.

Our calculations are based on Nåsell’s idea of transmission factors, as described by Bailey (1982). Transmission factors are analogous to reproductive numbers for a single “leg” of host-vector transmission (or heterosexual HIV transmission, see Dushoff et al. 2011). They give the number of new cases of one group that can be attributed to a single infectious individual of another group. In the case of malaria, the transmission factor from hosts to vectors ($\tau _{hv}$) is the average number of vector infections that are caused by a single infectious host, and the transmission factor from vectors to hosts ($\tau _{vh}$) is the average number of host infections that are caused by a single infectious vector. Unlike the reproductive numbers, we use these transmission factors only in the infinite-population limits. The reproductive number $\mathcal {R}_{0}$, for a vector-transmitted disease is equal to the product $\tau _{hv}$ $\tau _{vh}$, we call the ratio of the transmission factors $\rho = \tau _{hv}/\tau _{vh}$. For a directly transmitted disease, there is only one “transmission factor,” which we call $\tau$, and which is equal to $\mathcal {R}_{0}$.

Although we consider only homogeneous mixing here, in order to implement a model that takes finite-population size into account, we need to account for the fact that each infectious individual will create a discrete number of new infections. Since we assume that each infectious individual will have a constant contact rate over an exponentially distributed infectious period, the number of contacts is geometrically distributed. We then account for the fact that the population size is finite by allowing some of the potential infections to land on the same host.

## Methods

**Fig. 1:**
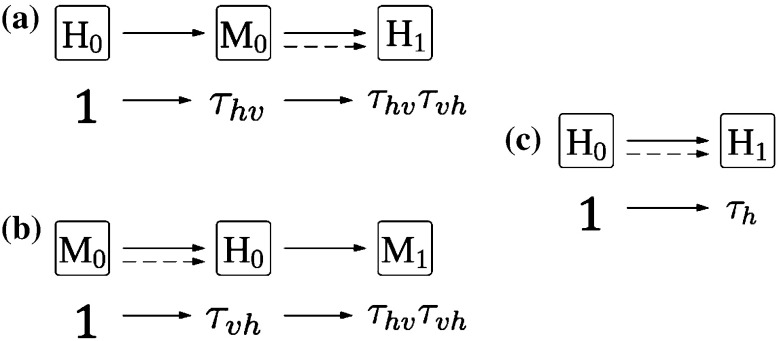
Schematic of one generation of disease transmission for **a**, **b** vector-borne and **c** directly transmitted diseases. The compartmental depiction represents transmission for both the infinite and the finite case. The *dashed arrows* indicate places where we change the calculation to take account of finite host populations. The depiction using transmission factors describes transmission only in the infinite case. **a** Transmission beginning with a single infectious host ($H_0$). That host goes on to produce $\tau _{hv}$ infected vectors ($M_0$) who each produce $\tau _{vh}$ new infected hosts resulting in $\tau _{hv}$ $\tau _{vh}$ new infected hosts ($H_1$) from a single infectious host. **b** Transmission beginning with a single infectious vector ($M_0$) who on average, infects $\tau _{vh}$ hosts ($H_0$). Each of those infectious hosts goes on to produce $\tau _{hv}$ new infected vectors, resulting in $\tau _{hv}$ $\tau _{vh}$ new infected vectors ($M_1$). **c** Direct transmission beginning with one infected individual ($H_0$) who infects an average of $\tau$ new individuals ($H_1$)

We calculate finite-population reproductive numbers for directly transmitted diseases, $\mathcal {R}(N)$, in a finite population of size $N$; and we calculate these finite-population reproductive numbers for vector-borne diseases, for host-to-host $\mathcal {R}(H)$, and vector-to-vector $\mathcal {Z}(H)$ transmission in a finite host population of size $H$ (under the assumption of an effectively infinite vector population). To calculate these finite-population reproductive numbers, we trace infections through one cycle of transmission. For a directly transmitted disease, hosts infect other hosts: we start with one “typical” infected individual and calculate how many individuals are infected by that individual. For a vector-borne disease, we look at cycles of transmission: for $\mathcal {R}(H)$, we start with one typical infected host and calculate how many vectors are infected from that host, and then how many hosts will become infected, on average, from that distribution of vectors; likewise for $\mathcal {Z}(H)$, we start with one infected vector, calculate how many hosts it infects and then how many vectors those hosts are expected to infect. These three scenarios $\mathcal {R}(N)$, $\mathcal {R}(H)$, and $\mathcal {Z}(H)$ are depicted diagrammatically in Fig. 1 for an infinite population, where the dashed arrows depict steps that change in the finite case.

**Fig. 2:**
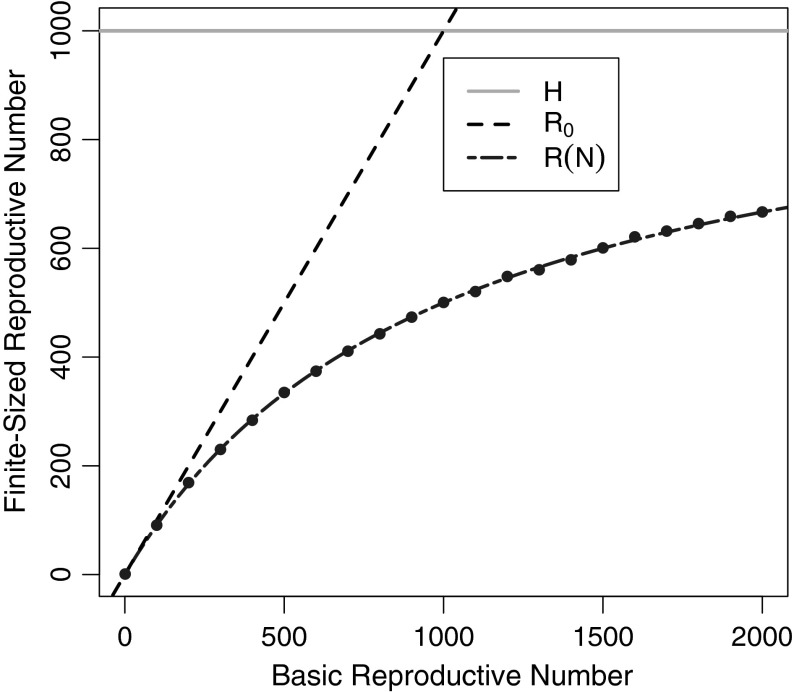
The finite-population reproductive number $\mathcal {R}(N)$ versus the basic reproductive number, $\mathcal {R}_{0}$, for directly transmitted diseases. Host population size is $N=1000$. The *blue points* represent the average of 1000 simulations each (Color figure online)

To validate our results, we simulated host-to-host and vector-to-vector transmission under the same assumptions used to calculate the finite-population reproductive numbers. Starting with a single infectious host (or vector), we simulate the number of hosts (or vectors) infected by those infectious individuals. We repeated those simulations 1000 times for a mosquito population of $M=100,000$, and took the mean of those 1000 simulations for each value of $\mathcal {R}_{0}$. Results are plotted in Figs. 2 and 4 (for direct- and vector-borne transmission, respectively). We explore the effects of smaller vector-population sizes in the appendix.

### Assumptions

For directly transmitted diseases, we assume a finite population of size $N$. Each infected host produces an average of $\tau$ potential new infections, using the geometric distribution, as discussed above; this is equivalent to assuming that the infection and recovery processes are Markovian. We assume that all hosts behave identically and independently. Since the host population is finite, some of these *potential* infections may fall at random on the same susceptible host, so the average number of *realized* infections in general, will be smaller.

In the case of vector-borne transmission, we assume that the host population is finite, of size $H$, and that the vector population is effectively infinite (i.e., much larger than the host population; we relax this assumption in the appendix) since mosquitoes are not the limiting factor. Thus, a single infected host produces a geometrically distributed number of new infections, with mean $\tau _{hv}$, in a susceptible vector population. We assume that all hosts and all vectors are identical and independent, as in the case of directly transmitted diseases. A single infected vector produces a geometrically distributed number of potential infectious events (we call these infectious *bites*) in the host population, with mean $\tau _{vh}$, however, because the host population is finite, some of these bites may fall at random on the same host, so the average number of new *infections* will be smaller.

### Calculation Framework

If we know that a generation of infected vectors produces $a$ potentially infectious bites on the finite host population, the probability that any individual host *escapes* infection is $\left( 1-\frac{1}{H}\right) ^a$. Thus, the expected number of new infections is $H\left( 1-\left( 1-\frac{1}{H}\right) ^a\right)$.

To calculate expectations, we use probability distributions over numbers of potentially infectious events $p(a)$, and corresponding generating functions, $\phi (x)=\sum _a p(a) x^a$. In particular, the generating function that corresponds to a geometric distribution with probability $P$ is:

1 $$\begin{aligned} \phi (x)= \sum _{a=0}^\infty (1-P)P^a x^a = \frac{1-P}{1-Px}. \end{aligned}$$

Since $\tau =\frac{P}{1-P}$ is the mean number of events, we can solve for $P$ to get $P=\tau /(\tau +1)$ and substitute to write $\phi (x) = \frac{1}{1+\tau (1-x)}$.

In particular, if $\phi _a(x)$ corresponds to the distribution of infectious bites on the host population $p(a)$, then we have that the expected number of infections, $I$, is:

2a $$\begin{aligned} I&= \sum _{a=0}^\infty H(1 - (1-1/H)^a) p(a) \end{aligned}$$

2b $$\begin{aligned}&= H\left( \sum _{a=0}^\infty p(a) - \sum _{a=0}^\infty (1-1/H)^a p(a)\right) \end{aligned}$$

2c $$\begin{aligned}&= H(1 - \phi _a(1-1/H)) \end{aligned}$$

## Results

### Calculation Framework

We use generating functions to calculate finite-population reproductive numbers for both directly transmitted and vector-borne diseases.

Since we assume that the number of infectious bites that land on a host is geometrically distributed, the generating function for the expected number of *bites* from one infectious vector is

3 $$\begin{aligned} \phi _{v1}(x)=\frac{1-P_{vh}}{1-P_{vh}x}, \end{aligned}$$

where $P_{vh}= \tau _{vh}/ (\tau _{vh}+ 1)$ is the probability that an infected vector bites a host.

If we substitute Eq. (3) into Eq. (2c), we have that the expected number of *infections* from one infectious vector is $I_1 = H-\phi _{v1}(1-1/H)$. Solving yields

4 $$\begin{aligned} I_1 = \frac{H \tau _{vh}}{H+\tau _{vh}}. \end{aligned}$$

Equation (3) gives the generating function for the number of infectious bites from one infectious vector, from this, we can calculate the generating function for the number of infectious bites by $m$ infectious vectors is

5 $$\begin{aligned} \phi _{vm}(x)=\left( \frac{1-P_{vh}}{1-P_{vh}x} \right) ^m \end{aligned}$$

Plugging in to (2), with mean $P_{vh}=\tau _{vh}/(\tau _{vh}+1)$, the distinct number of hosts infected by $m$ vectors is:

6 $$\begin{aligned} I_m = H\left( 1-\left( \frac{H}{H+\tau _{vh}}\right) \right) ^m \end{aligned}$$

### Direct Transmission, $\mathcal {R}(N)$

The expected number of infections that can be traced back to a single infected host is analogous to the expected number of bites from a single infectious vector in a finite population, above (for a vector-borne disease). Thus, by analogy with (4), the expected number of new infections from a single infectious host is

7 $$\begin{aligned} I_1= \frac{N \tau }{N + \tau }. \end{aligned}$$

Since $\mathcal {R}_{0}$ is exactly $\tau$, the expected number of infections from a single host is

8 $$\begin{aligned} \mathcal {R}(N)=\frac{N\tau }{N+\tau } = \frac{N \mathcal {R}_{0}}{N+\mathcal {R}_{0}} \end{aligned}$$

**Fig. 3:**
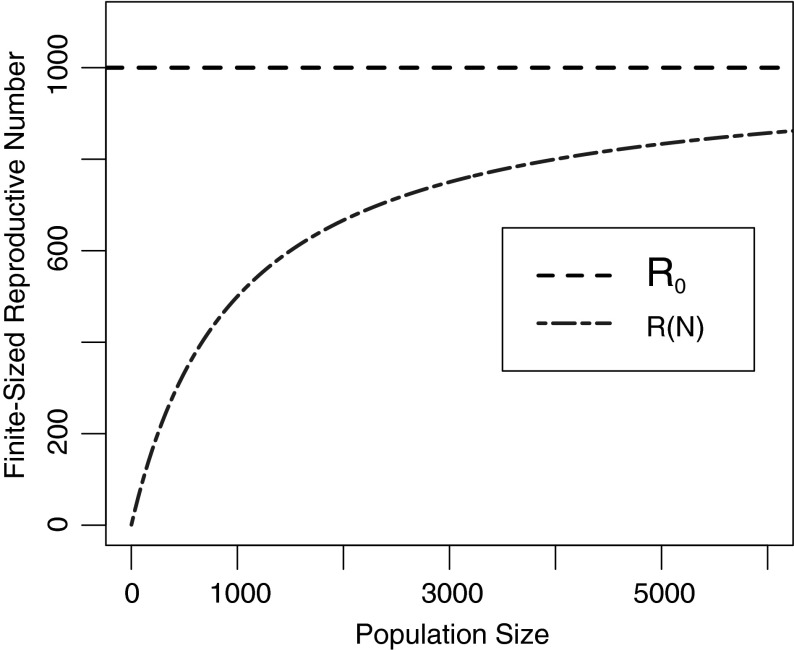
The finite-population reproductive number $\mathcal {R}(N)$ versus the population size, $N$, for directly transmitted diseases. The basic reproductive number is $\mathcal {R}_{0}=1000$ (Color figure online)

Figure 2 shows how our analytic calculation and simulations of $\mathcal {R}(N)$ increase with the basic reproductive number, for a fixed value of $H=1000$. $\mathcal {R}(N)$ diverges from $\mathcal {R}_{0}$ as the basic reproductive number approaches the population size—around $\mathcal {R}_{0}= 1/2 H$, and approaches the population size for very large values of $\mathcal {R}_{0}$. Simulated results match the analytically calculated results, as expected. Figure 3 shows how $\mathcal {R}(N)$ varies with $H$ for fixed $\mathcal {R}_{0}=1000$; $\mathcal {R}(N)$ converges on $\mathcal {R}_{0}$ as the population size increases relative to the basic reproductive number.

### Vector-Borne Disease Transmission

#### Vector-to-Vector Transmission, $\mathcal {Z}(H)$

To calculate the vector reproductive number, $\mathcal {Z}(H)$, we start with a single infectious vector, calculate the expected number of hosts infected by that single infectious vector, and then calculate the expected number of vectors infected by those infectious hosts.

Since we assume that the vector population is effectively infinite, (from (3)), we know that the number of hosts infected by a single infectious vector is

9 $$\begin{aligned} I_1 = \frac{H\tau _{vh}}{H + \tau _{vh}}. \end{aligned}$$

Those $I_1$ infected hosts go on to infect $\tau _{hv}$ vectors. Thus, the finite-population reproductive number for vectors is

10 $$\begin{aligned} \mathcal {Z}(H)=\frac{H\tau _{vh}\tau _{hv}}{H + \tau _{vh}} = \frac{H}{H+\tau _{vh}}\mathcal {R}_{0}\end{aligned}$$

#### Host-to-Host Transmission, $\mathcal {R}(H)$

To calculate the host reproductive capacity, $\mathcal{{R}}(H)$, we start with a single infectious host, calculate the expected number of vectors infected by that one host; then we calculate the expected number of hosts infected by infected vectors.

Starting from a single infected host, we calculate the expected number of vectors infected by that host. The number of infected vectors is distributed geometrically with mean $P_{hv}= \tau _{hv}/ (\tau _{hv}+1)$ where $P_{hv}$ is the probability that an infectious host infects a vector. Thus, the distribution of infected vectors (from a single infectious host) is

11 $$\begin{aligned} p(m)=(1-P_{hv})P_{hv}^m \end{aligned}$$

From (6), we know that the number of hosts infected by $m$ vectors is $I_m=H(1-(\frac{H}{H+\tau _{vh}}))^m$. The finite-population reproductive number for is $\mathcal{{R}}(H)=\sum _m p(m)I_m$. We calculate the finite-population reproductive number for hosts to be

12 $$\begin{aligned} \mathcal{{R}}(H) = \frac{\tau _{hv}\tau _mH}{H+\tau _{vh}\tau _{hv}+\tau _{vh}} = \frac{\mathcal {R}_{0}H}{\mathcal {R}_{0}+H+\tau _{vh}} \end{aligned}$$

**Fig. 4:**
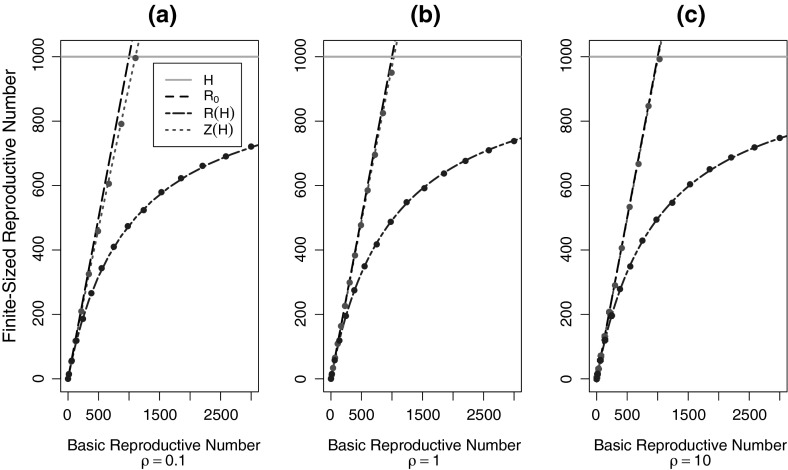
Plot of the basic reproductive number, $\mathcal {R}_{0}$ (*black*), versus the finite-sized reproductive numbers $\mathcal {R}(H)$ (*blue*) and $\mathcal {Z}(H)$ (*red*), for three values of $\rho$, for vector-borne diseases. The host population size, $H=1000$ (*gray*). We simulated the host-to-host reproductive number (*blue points*) and the vector-to-vector reproductive number (*red points*). For the simulations, the vector population size is $M=100,000$ (Color figure online)

**Fig. 5:**
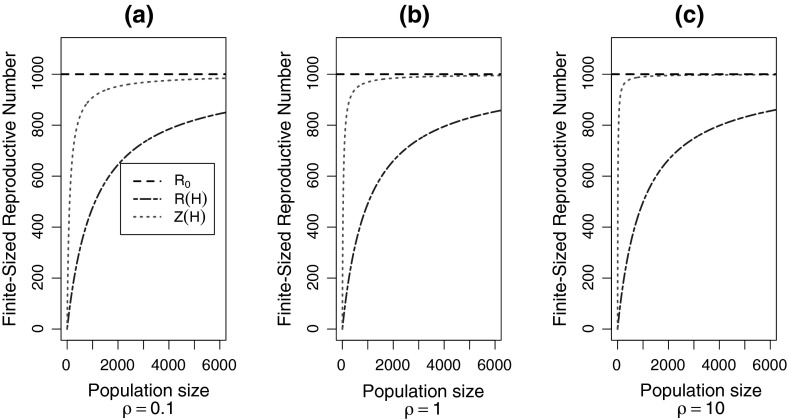
Plot of the population size $H$ versus the finite-population reproductive number, $\mathcal {R}(H)$ (*blue*) and $\mathcal {Z}(H)$ (*red*), for three values of $\rho$. The basic reproductive number, $\mathcal {R}_{0}=1000$ (*black*). **a** $\rho$= 0.1, **b** $\rho$= 1, and **c** $\rho$=10 (Color figure online)

The relationship between $\mathcal {R}(H)$, $\mathcal {Z}(H)$, and $\mathcal {R}_{0}$ is shown in Figs. 4 and 5, and the results are compared in Table 1. Figure 4 is a plot of the finite-population reproductive numbers ($\mathcal {R}(H)$ and $\mathcal {Z}(H)$) compared to $\mathcal {R}_{0}$ for a fixed population of size H. We varied $\tau _{hv}$ for three fixed values of $\rho$. It highlights the divergence of the host-to-host and vector-to-vector finite-population reproductive numbers and $\mathcal {R}_{0}$ as the reproductive numbers approach the size of the host population, $H$; which occurs at about $\mathcal {R}_{0}= H/3$. It also highlights the effect of $\rho$ on the divergence of $\mathcal {Z}(H)$ from $\mathcal {R}_{0}$, an effect better shown in Fig. 5.

**Table 1:** Finite-population reproductive number for direct- and vector-borne diseases compared with the basic reproductive number (for infinite population sizes)

Finite-sized reproductive numbers			Basic reproductive numbers	
Direct	Vector-borne		Direct	Vector-borne
$\mathcal {R}(N)$	$\mathcal {R}(H)$	$\mathcal {Z}(H)$	$\mathcal {R}_{0}$	$\mathcal {R}_{0}$
$\frac{N \tau }{N + \tau }$	$\frac{\tau _{hv}\tau _{vh}H}{\tau _{hv}\tau _{vh} + H + \tau _{vh}}$	$\frac{\tau _{hv}\tau _{vh}H}{ H + \tau _{vh}}$	$\tau$	$\tau _{hv}\tau _{vh}$

In addition, Fig. 4 shows the results of our simulations of vector-borne transmission in a finite-population. We simulated vector-to-vector and host-to-host transmission and plotted the resulting reproductive numbers. We see that our simulations match the analytically calculated results.

Figure 5 displays the relationship between the finite-sized reproductive numbers ($\mathcal {R}(H)$ and $\mathcal {Z}(H)$) and the infinite-population reproductive number, $\mathcal {R}_{0}$, for a fixed value of $\mathcal {R}_{0}$, varying the host population size, H, for three values of $\rho$. It shows $\mathcal {R}(H)$ and $\mathcal {Z}(H)$ converging on $\mathcal {R}_{0}$ as the size of the population increases and highlights the importance of $\rho$ in the convergence of $\mathcal {Z}(H)$ on $\mathcal {R}_{0}$. When hosts infect few vectors (relative to vectors infecting hosts), that is, when $\rho$ is small, Fig. 5a, $\mathcal {Z}(H)$ converges on $\mathcal {R}_{0}$ slower compared to when $\rho$ is large, Fig. 5c. $\rho$ has much less of an effect on the convergence of $\mathcal {R}(H)$.

## Discussion

Accurate calculation of the basic reproductive number, $\mathcal {R}_{0}$, is crucial for designing and implementing control and elimination programs. Smith et al. (2007) suggested that when $\mathcal {R}_{0}$ is large relative to the size of the population, as can happen with malaria, $\mathcal {R}_{0}$ does not accurately reflect the disease dynamics. They introduced the idea of finite-population reproductive numbers for both host and vectors, for malaria, and used simulations to estimate both $\mathcal {R}_{0}$ and the finite-population reproductive numbers ($\mathcal{{Z}}_0(H)$ and $\mathcal{{R}}_0(H)$), while allowing for heterogeneous biting, transmission-blocking immunity, and sampling issues.

Here, we consider the simpler case of homogeneous mixing, where all hosts are equally attractive to mosquitoes, and we are able to derive analytic formulas for those finite-population reproductive numbers for both vector-borne ($\mathcal {Z}(H)$ and $\mathcal {R}(H)$) and for directly transmitted diseases ($\mathcal {R}(N)$). We find simple formulas for these quantities in terms of transmission factors ($\tau _{hv}$, $\tau _{vh}$, and $\tau$) (Bailey 1982) (Table 1), and show that the finite-population reproductive numbers, particularly $\mathcal {R}(N)$ and $\mathcal {R}(H)$, diverge from $\mathcal {R}_{0}$ when $\mathcal {R}_{0}$ approaches the population size. We then simulated our results in a finite population of hosts to validate our analytic calculations (Figs. 2, 4).

Since we assume a finite-population size in the direct transmission case, and a finite host population size in the vector-borne case (with an infinite vector population size), the reproductive numbers $\mathcal {R}(N)$ and $\mathcal {R}(H)$ are necessarily smaller than the size of the population, however, for vector-borne transmission, $\mathcal {Z}(H)$ can exceed the host population size as the vector population is infinite (Figs. 4, 5). However, unlike $\mathcal {R}_{0}$, $\mathcal {Z}(H)$ does decrease as it approaches the population size, but not as quickly as $\mathcal {R}(H)$ does (Figs. 4, 5). Our results show that $\mathcal {Z}(H)$ is very similar to $\mathcal {R}_{0}$ when $\tau _{hv}$ is large relative to $\tau _{vh}$ (large $\rho$) (Figs. 4c, 5c), and somewhat smaller (though still larger than $\mathcal {R}(H)$) when $\tau _{hv}$ is small relative to $\tau _{vh}$ (small $\rho$) (Figs. 4a, 5a). In other words, the number of vectors infected by a single infectious vector is more strongly affected by the host population size, when the number of *hosts* infected by a single vector is large compared to the number of *vectors* infected by a single host.

If we were to relax the assumption of infinite vector population size, both $\mathcal {R}(H)$ and $\mathcal {Z}(H)$ would be limited by $M$ in an analogous way to how they are limited by $H$: $\mathcal {R}(H)$ would decrease if $M$ was small relative to the reproductive number (in the same way that $\mathcal {Z}(H)$ decreases as a result of $H$) but it would not be limited by $M$; and $\mathcal {Z}(H)$ would be bounded by $M$ (just as $\mathcal {R}(H)$ is bounded by $H$).

Since $\mathcal {R}(N)$ and $\mathcal {R}(H)$ are bounded by the population size and $\mathcal {R}_{0}$ is not, they diverge. In the case of directly transmitted diseases, $\mathcal {R}(N)$ diverges from $\mathcal {R}_{0}$ near $\mathcal {R}_{0}=N/2$ (Fig. 2). Similarly, for vector-borne diseases, $\mathcal {R}(H)$ diverges much sooner than $\mathcal {R}(N)$ (for directly transmitted diseases). Our results show that when $\mathcal {R}_{0}$ is near the size of the host population size, $\mathcal {R}_{0}$ overestimates the actual dynamics, making control and elimination seem more difficult then they actually are.

It is well known that malaria-transmitting mosquitoes prefer some hosts over others for a variety of reasons (for example Knols et al. 1995; Mukabana et al. 2002). This heterogeneity has complicated effects in simple models, it increases the reproductive number (Woolhouse et al. 1997; Dye and Hasibeder 1986), but it can decrease finite-population reproductive numbers (Smith et al. 2007). Extending the work here to gain analytic insight into finite-population reproductive numbers in the presence of heterogeneity in host attractiveness to mosquitoes would be a valuable contribution to understanding diseases spreading in small populations.

## Figures and Tables

**Fig. 6:**
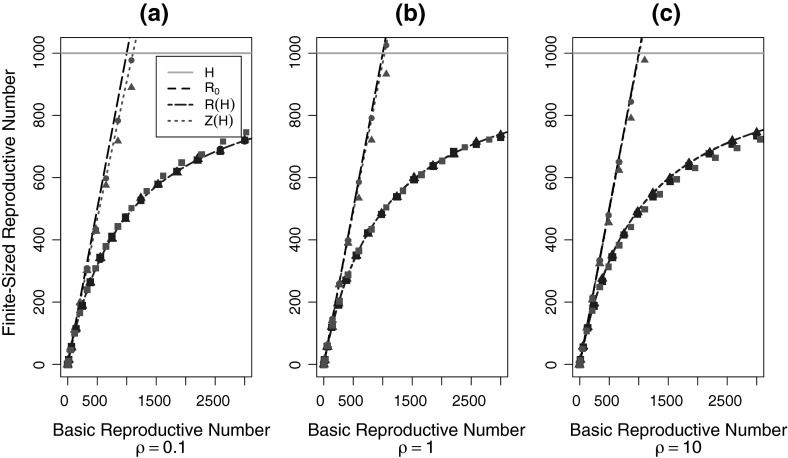
The basic reproductive number, $\mathcal {R}_{0}$ (*black*), versus the finite-sized reproductive numbers $\mathcal {R}(H)$ (*blue*) and $\mathcal {Z}(H)$ (*red*), for three values of $\rho$, for vector-borne diseases. The host population size, $H=1000$ (*gray*). Points represent the average of 1000 simulations. We vary the vector population size, from $M=100,000$ (*closed circles*) to $M=10,000$ (*triangles*), to $1,000$ (*squares*). **a** $\rho$= 0.1, **b** $\rho$= 1, and **c** $\rho$=10 (Color figure online)

## Appendix: Finite Vector Population

We assume for the calculations of $\mathcal {R}(H)$ and $\mathcal {Z}(H)$that the population of vectors is effectively infinite. In our simulations of finite-sized reproductive numbers, we chose a population of vectors to be very large relative to the host population size. However, here we explore how finite vector population size affects the simulations of the finite-population reproductive numbers (Fig. 6).

We find that the vector-population size, M, does not significantly affect the finite-sized reproductive numbers unless $M$ is small relative to the host population size, $H$. We also see that when $M$ is small relative to $H$, $\rho$ has the opposite effect on $\mathcal {Z}(H)$ than when the vector population size is infinite: when $\rho$ is small, for the same value of $M$, $\mathcal {Z}(H)$ diverges from $\mathcal {R}_{0}$ slower than when $\rho$ is large. That is, when hosts are better at transmitting to vectors (than vectors are at transmitting to hosts) the effect of small vector-population size is smaller than when the vectors are better at transmitting to hosts (relative to hosts transmitting to vectors).
